# Blunted perception of breathlessness in three cases of low grade insular-glioma

**DOI:** 10.3389/fnins.2024.1339839

**Published:** 2024-02-12

**Authors:** Tom P. Chapman, Sarah M. Farrell, Puneet Plaha, Alexander L. Green, Shakeeb H. Moosavi

**Affiliations:** ^1^Department of Biological and Medical Sciences, Oxford Brookes University, Oxford, United Kingdom; ^2^Department of Clinical Neurosciences, John Radcliffe Hospital, Oxford, United Kingdom; ^3^Nuffield Department of Surgical Sciences, University of Oxford, Oxford, United Kingdom

**Keywords:** dyspnoea, hypercapnia, air hunger, insular cortex, neurosurgery, brain tumour, short of breath

## Abstract

Better understanding of breathlessness perception addresses an unmet clinical need for more effective treatments for intractable dyspnoea, a prevalent symptom of multiple medical conditions. The insular-cortex is predominantly activated in brain-imaging studies of dyspnoea, but its precise role remains unclear. We measured experimentally-induced hypercapnic air-hunger in three insular-glioma patients before and after surgical resection. Tests involved one-minute increments in inspired CO_2_, raising end-tidal *P*CO_2_ to 7.5 mmHg above baseline (38.5 ± 5.7 mmHg), whilst ventilation was constrained (10.7 ± 2.3 L/min). Patients rated air-hunger on a visual analogue scale (VAS). Patients had lower stimulus–response (2.8 ± 2 vs. 11 ± 4 %VAS/mmHg; *p* = 0.004), but similar threshold (40.5 ± 3.9 vs. 43.2 ± 5.1 mmHg), compared to healthy individuals. Volunteered comments implicated diminished affective valence. After surgical resection; sensitivity increased in one patient, decreased in another, and other was unable to tolerate the ventilatory limit before any increase in inspired CO_2_.We suggest that functional insular-cortex is essential to register breathlessness unpleasantness and could be targeted with neuromodulation in chronically-breathless patients. Neurological patients with insula involvement should be monitored for blunted breathlessness to inform clinical management.

## Introduction

James Parkinson stated in his seminal paper on the ‘shaking palsy’ in 1817 that the patient ‘*seemed to catch their breath rather hard*’ (Parkinson, 2002) yet monitoring of breathlessness in management of neurological patients is rare. Dyspnoea is a complaint of seriously ill hospitalised patients, especially those with cardiopulmonary conditions (Edmonds et al., 2001; Hutchinson et al., 2018). Within the Nuffield department of surgical sciences at Oxford, we noticed cases of new-onset breathlessness, or relief of existing breathlessness, before and after brain surgery. The insular-cortex of the limbic system has been universally reported to be predominantly activated in functional brain-imaging studies of experimentally-induced breathlessness in healthy individuals, insinuating a principal role in breathlessness perception (Banzett et al., 2000; Evans et al., 2002). To progress our understanding of the neural mechanisms of breathlessness, we applied established techniques (Grogono et al., 2018) to test breathlessness sensitivity in patients with low-grade insula-gliomas, before and after surgical resection of the gliomas. We focused on air-hunger (AH) defined as an uncomfortable urge to breathe which is a particularly unpleasant component of clinical dyspnoea (Banzett et al., 2008).

## Materials and methods

Three consecutive cases of low-grade insula-glioma (two males, 41 ± 4 years., 181 ± 13 cm height, and 82 ± 40 kg weight at pre-surgery testing) awaiting surgery, were selected. Patients attended a single test session before and at least 5 months after surgical resection of their glioma. NRES South Central-Oxford C provided ethics approval (REC ref.: 11/SC/0229), and patients gave written informed consent.

### Experimentally induced air hunger

**Figure 1:**
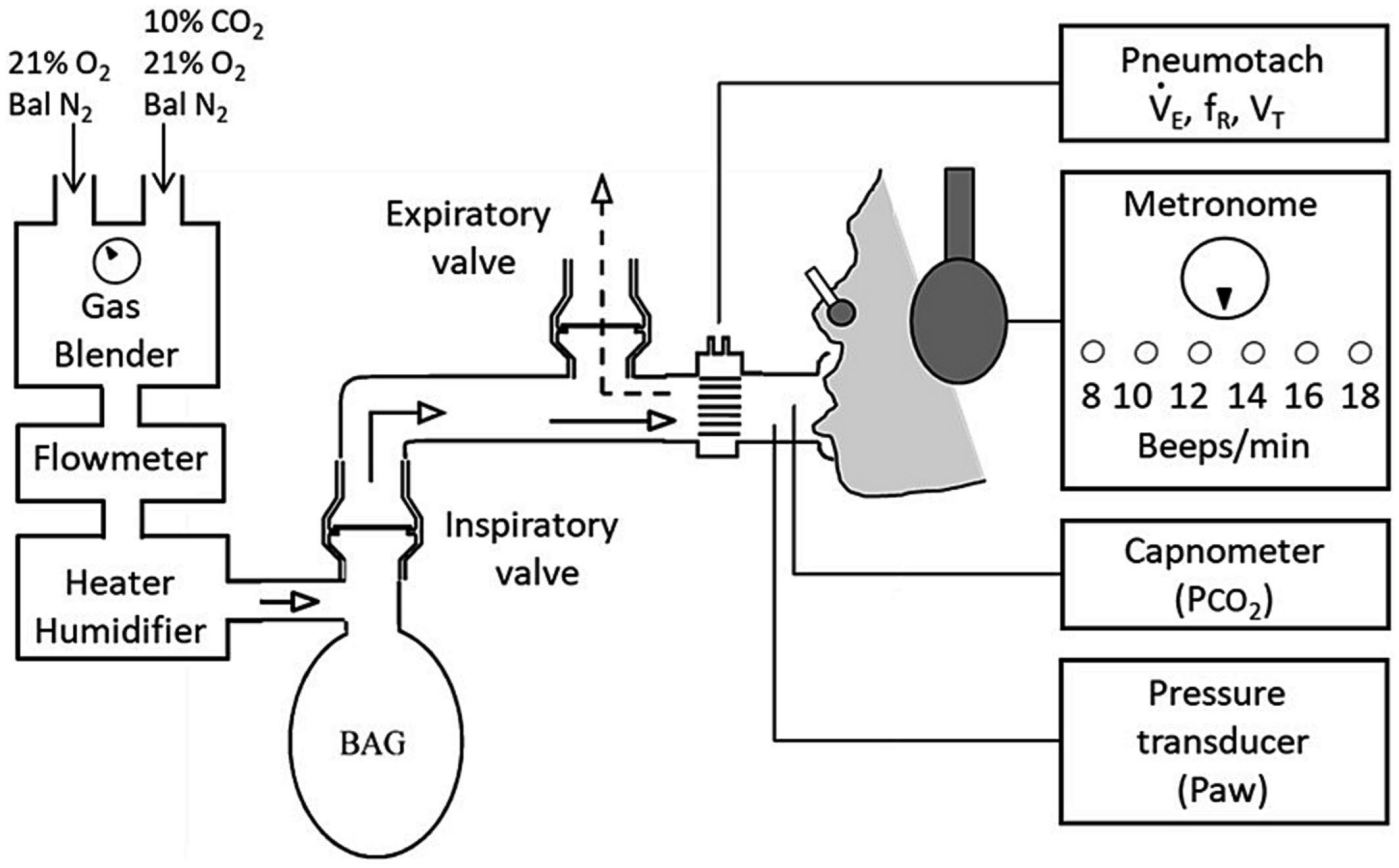
Experimental setup and protocol. Experimental setup: Participants breathed via a mouthpiece from a 3 L anaesthetic bag into which the flow of fresh gas was set to the participants’ baseline minute ventilation (V_E_). A Metronome was used to set breathing frequency (f_R_) to the participants’ spontaneous rate at baseline.

Hypercapnia was used with constrained ventilation to induce AH, an established experimental model for inducing the AH component of clinical dyspnoea (O’donnell et al., 2013; Banzett et al., 2021). Participants breathed via a circuit that separated inspiration from expiration (Figure 1). An inspiratory reservoir provided the only source of inspiration (3-litre anaesthetic bag). A fixed flow of humidified air was fed into the bag to match resting ventilation, and patients breathed in time with a metronome to match respiratory frequency, thus fixing tidal volume. To induce AH, up to 7% CO_2_ was added to the bag using an air-oxygen blender supplied by gas cylinders containing 10%CO_2_ in air, and medical air. Participants rated AH using a 10cm visual analogue scale (VAS), cued by a LED every 15s.

### Study design

An initial familiarisation hypercapnic ‘ramp’ test involved 1-min increments of 1.3% inspired CO_2_ that raised end-tidal *P*CO_2_ (P_ET_CO_2_) by between 2 and 3 mmHg/min whilst participants rated any breathing discomfort. Extremes of the VAS were labelled ‘none’ (no breathing discomfort) and ‘extreme’ (the tolerable limit). End-point was when tolerance was reached and participants came off the mouthpiece or P_ET_CO_2_ reached 60 mmHg. Debriefs involved; (i) asking patients to describe their breathing when they had rated high, (ii) presenting a list of respiratory descriptors for participants to select the three most applicable to ensure that they could distinguish AH (i.e., urge to breathe, shortness of breath, feeling of suffocation/smothering). Following debrief, the test was repeated with participants instructed to exclusively rate AH.

### Data processing and analysis

Analogue signals were digitised and recorded for offline analysis (Spike 2, Cambridge Electronic Design, United Kingdom). Breath-by-breath P_ET_CO_2_ was processed with a 60s moving average to account for delay in AH response dynamics (Banzett et al., 1996). AH ratings were plotted against change in P_ET_CO_2_ from that at AH-threshold determined by visual inspection of individual scatter plots.

The pooled scatter for the stimulus response data from 10 healthy individuals (mean ± sd age 25 ± 3 years, height was 169 ± 12 cm, and weight was 70 ± 23 kg) was extracted from a previous study using the same protocol (Grogono et al., 2018) and plotted alongside the stimulus response data for the patients to determine the overlap in scatter. The x-axis was normalised for the *P*CO_2_ at individual AH thresholds to align the individual stimulus response slopes. The AH threshold was determined by visual inspection of individual stimulus response curves (see Supplementary Figure 2). As no AH threshold was evident for case 1, the average AH threshold in the other two patients was assumed in order to include the data points for case 1 in this plot.

Individual air hunger sensitivities were also determined by linear regression through individual stimulus response scatter normalised for AH threshold. The average AH sensitivity (slope) between patients (*n* = 3) and healthy controls (*n* = 10) was compared using an unpaired *t*-test. The same *t*-test was also applied to the comparison of the group mean AH threshold for each group.

### Brain imaging processing and analysis

**Figure 2:**
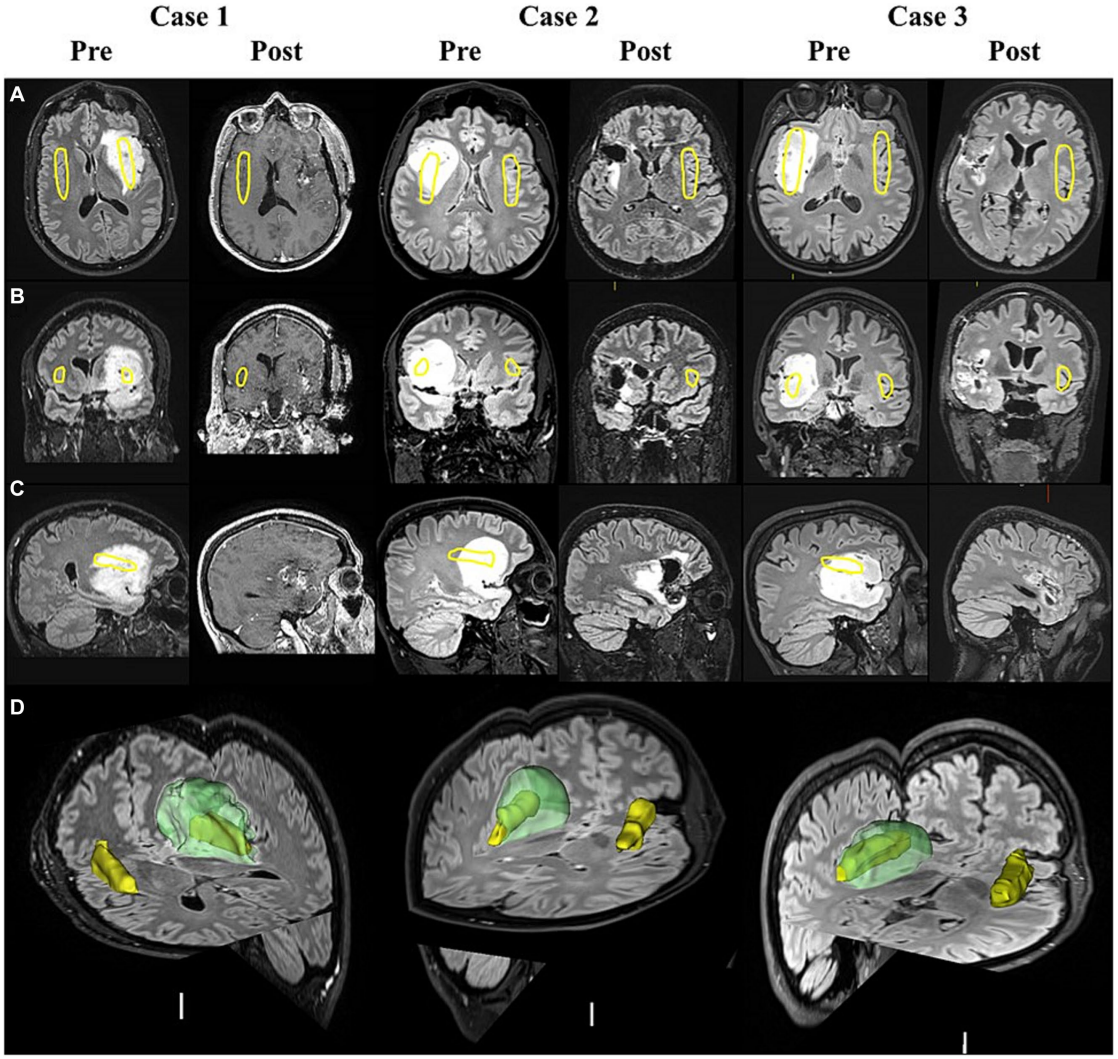
MRI scans of insula-glioma pre- and post-surgery. T2 weighted FLAIR MRI images displayed in the axial **(A)**, coronal **(B)**, and sagittal **(C)** planes before surgery (Pre) and after surgery (Post) for cases 1 to 3. Case-1 did not have a post-surgery FLAIR scan as they were isolating abroad during the COVID-19 pandemic and therefore available post-surgery T1 weighted MRI scans were used (48 months before post-surgery testing). Yellow outlines represent the positioning of the insular cortex. Panel **(D)** displays the segmented insula (yellow) and glioma (Green) generated using pre-surgery FLAIR scans in 3D slicer for each case.

T2-weighted FLAIR MRIs were imported into 3D-Slicer (Fedorov et al., 2012). A semi-automated segmentation editor within 3D-slicer was used to generate 3D images of the glioma and generate reports on its volume, shown to be an accurate alternative to manual segmentation by experienced consultants (Egger et al., 2013). The insular cortex was manually segmented on the healthy hemisphere and subsequently flipped to generate bilateral-segmented insular, assuming symmetry (Figure 2). Glioma details were obtained from clinical notes. Pre-surgery and post-surgery FLAIR scans were acquired within 1 year and 1 month of surgery, respectively. Clinical notes specified there was no change in size of glioma from the between pre-surgery scan and surgery.

## Results

Interval between diagnosis and surgical resection averaged 7 ± 1 months, and that between pre and post-surgery testing was 48, 9 and 7 months, respectively (Table 1). Demographics and pre- and post-MRI scans are provided in Table 1 for each case. Post-surgery weight and medications for each case were as follows: Case 1; 111 kg, Keppra. Case 2; 65 kg, Keppra. Case 3; 47 kg, Lacosamide.

**Table 1:** Patient demographics and clinical details.

Case	Age (yrs)	Sex (M/F)	Ht (cm)	Wt (kg)	Medications	Type and site of LGG	LGG volume (cm^3^)	Post-surgery report
1	45	M	195	127	Lamotrigine75 mg 2/day	Oligodendroglioma of the right temporal insula extending to amygdala	82	Majority of tumour resected. No follow up post-surgery scans near to date of testing.
					2				38	M	170	67	Keppra1,000 mg	Left frontal insula astrocytoma	42	Maturation of resection cavity involving left inferior frontal gyrus and lateral aspect of left temporal pole. Compared with the healthy hemisphere; ~60% of glioma cavity appears normal, suggesting de-compression of insula tissue
3	41	F	178	52		Keppra125 mg, Cyclizine, Insulin, Lacosamide 50 mg Clobazam 10 mg.	Left astrocytoma centred in left insula	54					Maturation of resection cavity. Emergence of tissue within the resection cavity which does not match healthy hemisphere. Entire insula seems abnormal compared to the unaffected hemisphere.			

LGG, Low Grade Glioma.

**Figure 3:**
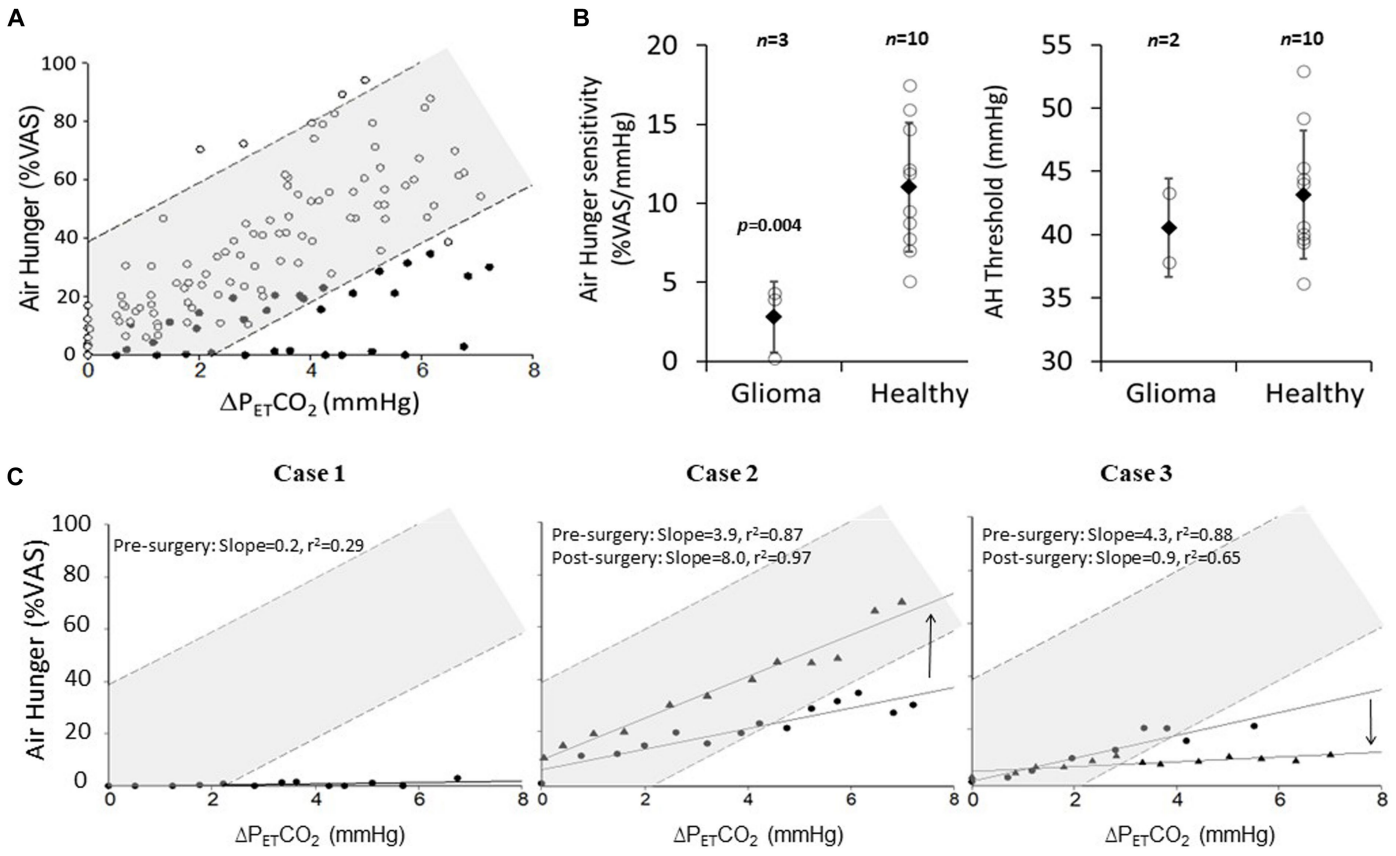
The effect of low-grade insula-glioma and its removal on air hunger (AH) sensitivity. **(A)** Pooled AH VAS ratings plotted against change in P_ET_CO_2_ above threshold AH for 3 patients with insula-glioma (closed circles) and 10 healthy-controls (open circles). Breath-by-breath P_ET_CO_2_ was processed with a 60 s moving average to account for delay in AH response dynamics (5). AH threshold was determined visually from individual slopes. Light shading shows 95% prediction intervals. Normative data was extracted from a previous publication using the same protocol (3). **(B)** Mean ± sd (closed diamonds) of individual AH sensitivities (left) and thresholds (right) for pre-surgery glioma patients (*n* = 3 for left and *n* = 2 for right) and healthy controls (*n* = 10). Individual scatter around these means are also shown (open circles). Average AH sensitivity was lower in patients (^*^p = 0.004; unpaired Student’s *t*-test). **(C)** AH plotted against change in P_ET_CO_2_ above AH threshold for pre-surgery (circles) and post-surgery (triangles). Case-1 (left) did not rate any AH pre-surgery, the threshold was assumed to be the average of Cases-2 and 3 for inclusion in this figure. Case 1 could not tolerate the post-surgery test. 95% prediction and 95% confidence intervals for the pooled healthy control data is represented on these graphs by the darker and lighter shaded regions. Direction of arrows for case 2 and 3 represent whether AH sensitivity has increased or decreased.

Figure 3A shows that there is little overlap between the scatter of the pooled stimulus–response data from healthy controls and that of the 3 patients. Mean ± sd individual sensitivity was significantly lower in patients pre surgery (2.8 ± 2 vs. 11.0 ± 4 %VAS/mmHg; *p* = 0.004, students unpaired *t*-test; Figure 3B, left). However, the group difference in AH thresholds did not achieve statistical significance (Figure 3B, right). After surgery, Case-2 recovered some AH sensitivity (3.9 to 8.0%VAS/mmHg), returning to within the 95% predicted normal range (Figure 3C, middle). Case-3 had a substantial further reduction in AH sensitivity (4.3 to 0.9%VAS/mmHg; Figure 3C, right). Since Case-1 did not rate any AH during the pre-surgery test, we assumed an AH threshold as the average of that seen in Case-2 and Case-3 in order to plot Case-1 in Figure 3C (left). We could not do the same for the post-surgery data in Case-1 because they terminated the test before any change in P_ET_CO_2_.

## Discussion

Low-grade insula-glioma appears to blunt AH sensitivity without rightward shift in threshold, and its resection can increase or reverse this effect. Gliomas can compress, displace, and infiltrate brain tissue; generally, without destroying grey or white matter or eliciting neurological deficits (Whittle, 2004). However, blunted AH implies damage to the insula’s neural fabric with a hidden functional deficit only manifested if breathlessness pre-exists or is measured.

The insular receives a multitude of visceral afferents (Gogolla, 2017), is activated in brain-imaging studies of unpleasant sensations including pain (Duerden and Albanese, 2013) and breathlessness (Banzett et al., 2000; Evans et al., 2002), and if lesioned, results in alterations in pain (Starr et al., 2009). One previous study compared the dyspnoea associated with inspiratory resistive loading between four patients with ischaemic stroke of the insula, and healthy controls (Schon et al., 2008). A reduction in intensity and unpleasantness of dyspnoea was reported in the patients. However, this conclusion was unconvincing as it was based upon change from baseline dyspnoea to the dyspnoea with applied respiratory stimulus, and the patients had elevated baseline levels of dyspnoea (Adams et al., 2009). In contrast none of our patients rated any AH at normocapnia which might suggest that glioma and ischaemia of the insular may have different impact on its functionality. Our data on the other hand, provides clear evidence that a more unpleasant form of dyspnoea (air hunger) is reduced in patients with insular glioma.

### Possible mechanism of blunted perception

The fact that we did not observe a significant difference in AH threshold may imply that there is no impact of the insular glioma on the gating of the AH signal which is thought to take place in the thalamus or brainstem. However, the blunted sensitivity may imply that the neural processing of this signal at the insular has been affected. We discuss below several possible explanations for complete absence of AH sensitivity pre-surgery in Case-1, compared to diminished sensitivity in Cases 2 and 3:

- Case-1 glioma engulfed the entire right insular whereas the tip of the left posterior insular extended beyond the glioma in Cases 2 and 3 (Table 1). This could account for some preserved AH sensitivity in cases 2 and 3. Pain research suggests that sensory-intensity is processed in posterior, and sensory-affect in anterior insular (Gogolla, 2017). By this token, AH with high affective valence is more likely to associate with anterior insular function consistent with brain imaging studies. However, involvement of anterior insular was not different between case 1 and the others.
- A more likely explanation for the complete absence of AH sensitivity in Case-1 is that they had right-sided glioma. Brain-imaging of dyspnoea showed greater activity in the right insular (Banzett et al., 2000). We cannot discount that Case-1 had naturally elevated AH threshold, but most healthy individuals rate close to full-scale at the highest hypercapnia we imposed (49 mmHg; Banzett et al., 1996).
- The glioma in Case-1 extended into the amygdala, which is linked to fear, anxiety, and breathlessness in COPD (Esser et al., 2017). After the pre-surgery test, Case-1 stated; *‘I knew my breathing was not right, but it did not bother me at all’*, suggesting specific impact on the affective domain. This is consistent with absent hypercapnia induced dyspnoea in patients where epileptic seizures extending to the amygdala and in two cases of focal electrical stimulation (50 Hz) of the amygdala (Dlouhy et al., 2015).
- Glioma in Case-1 was an oligodendroglioma and an astrocytoma in the others. We speculate whether disruption of neuronal insulation by the oligodendroglioma has greater impact on sensitivity than disruption of the supply of nutrients and structural support by astrocytomas.

### Effect of surgical resection on AH sensitivities

Post-surgery, Case-1 terminated the test before any inspired CO_2_ load. We suspect that during the post-surgery test ventilatory constraint acted as a cue for intolerance, consistent with their debrief comment; ‘*I cannot breathe, so obviously I am breathless’*. Recovery of AH-sensitivity post-surgery in Case-2 (Figure 3C, middle) may reflect cross-hemispheric neuroplasticity with heightened function of the unaffected right-insular during the 9 months between pre and post-surgery testing. We cannot exclude the possibility that some residual functioning insular may have survived resection, but since most of the left-insular was removed neuroplasticity of the right insular is more likely responsible for recovery of sensitivity. The further reduction in AH sensitivity post-surgery in Case-3 is supported by the fact that this patient, who had resting breathlessness before surgery, stated that after surgery this had ‘*completely disappeared*’ and ‘*breathing now feels very easy, as if the air is lighter’*. Although neuroplasticity of unaffected insular could occur within 5-months (Duffau, 2020), Case-3 had no improvement despite 7-months between testing. This patient had further reduction in sensitivity post-surgery, we propose that residual functioning insular tissue in the glioma was lost in resection.

### Clinical implications

The belief that low-grade gliomas (whether compressive or infiltrative) are not commonly associated with neurological deficit may need to be re-visited based on our findings that the ability to sense unpleasant symptoms such as air hunger may be lost. This may otherwise be classed as ‘no neurological deficits’ unless specific testing is conducted, or disappearance of pre-existing symptoms are observed.

Insular lesions are commonly associated with dysphagia. Blunted breathlessness perception alongside this may compromise patients’ ability to detect aspiration-pneumonia, pulmonary embolism, and upper-respiratory tract infections delaying treatment and exacerbating the condition. Post-mortem records of 30 patients with pulmonary embolism after stroke (Wijdicks and Scott, 1997), revealed sudden-death had occurred in half the patients but pleuritic pain and dyspnoea were major symptoms only in the others. A hitherto unexplored possibility is that blunted dyspnoea played a role in the fatal outcome of the half who died suddenly, consistent with a suggested decrease in symptom perception in patients with a history of near fatal asthma attacks (Kikuchi et al., 1994). We also cannot comment on whether blunted AH sensitivity is specific for insular-glioma or whether other sensory modalities are affected.

### Limitations

This case series is limited primarily in the small number of cases we were able to study. We compared the individual stimulus responses in these three cases with that of 10 healthy control datasets extracted from a previous study from our lab (Grogono et al., 2018). Since we only have three cases our use of an unpaired *t*-test to compare the mean AH sensitivity and threshold between groups (Figure 3B) may be deemed inappropriate even though the data passed the Shapiro Wilks test of normality. Putting aside the statistics that we have presented, the individual scatter for the AH sensitivities in the healthy individuals does not overlap with that of the corresponding scatter for the patients (Figure 3B, left).

The patient group was older, taller and heavier on average than the healthy controls. To check if this difference in group demographics could account for the group difference in average slope, we selected the 3 healthy controls who were closest matched with respect to sex, age, height, weight and BMI. The revised average height, weight and BMI compared well between groups: 181 vs. 181 cm, 82 vs. 90 kg, 26.8 vs. 24.3 kg/m^2^, respectively. To check if the AH slopes in these better-matched healthy controls remain substantially steeper than that in the patients, we re-plotted Figure 3A with just the 3 best-matched healthy controls (Supplementary Figure 1); this revised figured showed that the pooled healthy control data had an even steeper response slope (15.5 %VAS/mmHg).

Age however remained significantly younger for the healthy controls when only including the three best-matched controls (27 vs. 41 years; *p* = 0.009, unpaired two-tailed *t*-test). We cannot exclude the possibility that age could have an effect in reducing the slope of the hypercapnic air hunger response -however this is unlikely based on previous studies that have reported slopes of dyspnoea responses in older individuals using the same experimental model (hypercapnia with constrained ventilation). For example, the oldest participant in the study by Banzett et al. (2021) was 57 years old (older than our oldest patient) and yet they had the second highest response slope (9.4 mm/mmHg).

## Conclusion

In conclusion, glioma of the insular cortex blunted breathlessness, resonating with reports of insular activation in brain-imaging studies. The differential effects of surgical resection on breathlessness sensitivity may be explained by compensatory increases in insular activity in the healthy hemisphere over time, and acute loss of residual functioning tissue by resection. This case-series advocates; (i) monitoring breathlessness in patients with insular-glioma, (ii) targeting insular with neuromodulation for symptomatic relief of intractable dyspnoea in cardiopulmonary conditions.

## Data availability statement

The raw data supporting the conclusions of this article will be made available by the authors, without undue reservation.

## Ethics statement

The studies involving humans were approved by NRES South Central Oxford C (REC ref.:11/SC/0229). The studies were conducted in accordance with the local legislation and institutional requirements. The participants provided their written informed consent to participate in this study. Written informed consent was obtained from the individual(s) for the publication of any potentially identifiable images or data included in this article.

## Author contributions

TC: Data curation, Formal Analysis, Funding acquisition, Methodology, Project administration, Resources, Software, Writing – original draft, Writing – review & editing, Investigation. SF: Data curation, Investigation, Project administration, Supervision, Writing – original draft, Writing – review & editing, Methodology. PP: Resources, Supervision, Writing – review & editing, Conceptualization, Methodology. AG: Conceptualization, Investigation, Resources, Supervision, Writing – review & editing. SM: Conceptualization, Formal Analysis, Investigation, Methodology, Resources, Supervision, Writing – review & editing, Funding acquisition, Project administration.

## Supplementary material

SUPPLEMENTARY FIGURE 1 Comparison of stimulus response for patients and best matched controls (*n*=3). Pooled AH VAS ratings plotted against change in PETCO2 above threshold AH for 3 patients with insula-glioma (closed circles) and 3 best matched healthy-controls (open circles). Solid lines are linear regression with 95% CI (dashed lines) and 95%PI (dotted lines). For the patient group the data from case 1 was not included in the linear regression because they rated close to 0%VAS AH throughout. Had we included the data for case 1 in the linear regression the average slope for the patient group would have been even more shallow compared to the best matched healthy controls.

SUPPLEMENTARY FIGURE 2 Individual hypercapnic air hunger response slopes. For the patient group, AH responses for both pre-surgery (closed circles) and post-surgery (triangles) are included. Open circles are used for the healthy controls.
