# Supplementary figures and images for: Blunted perception of breathlessness in three cases of low grade insular-glioma
Source: Front Neurosci. 2024 Feb 12;18:1339839. doi: 10.3389/fnins.2024.1339839 (PMC10894922; doi:10.3389/fnins.2024.1339839)

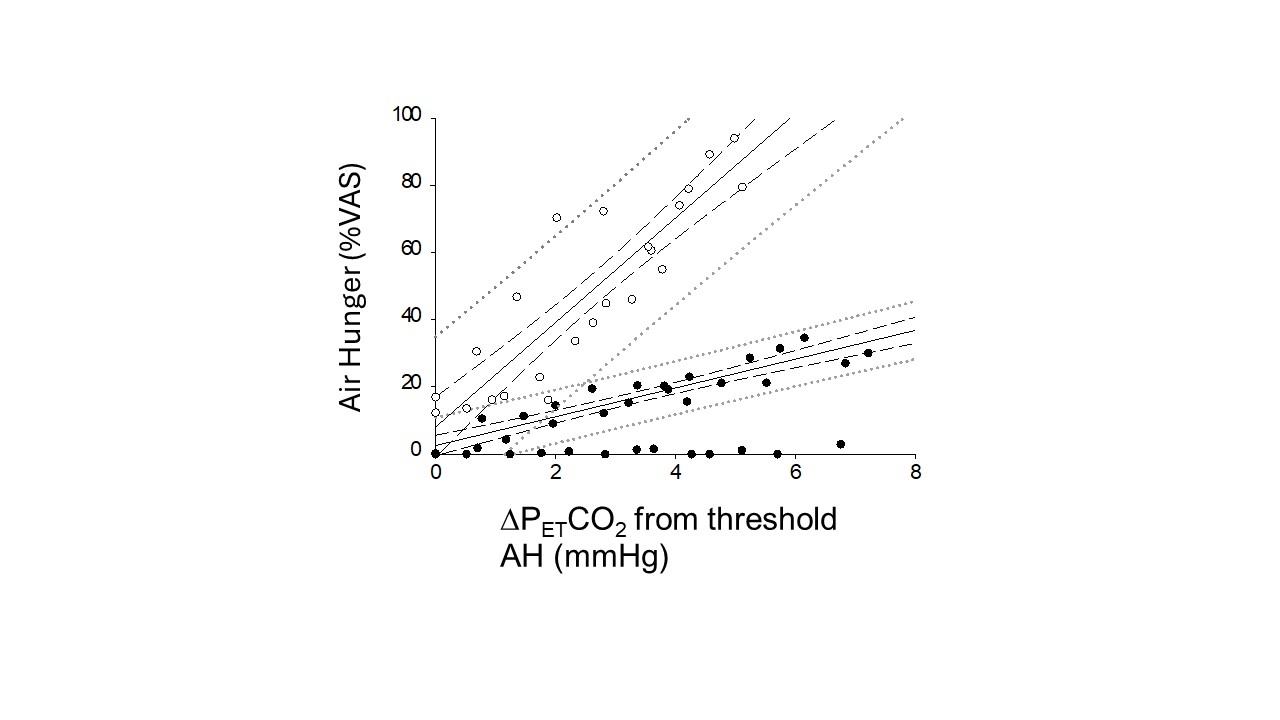

Supplement: SUPPLEMENTARY FIGURE 1 — Comparison of stimulus response for patients and best matched controls (n=3). Pooled AH VAS ratings plotted against change in PETCO2 above threshold AH for 3 patients with insula-glioma (closed circles) and 3 best matched healthy-controls (open circles). Solid lines are linear regression with 95% CI (dashed lines) and 95%PI (dotted lines). For the patient group the data from case 1 was not included in the linear regression because they rated close to 0%VAS AH throughout. Had we included the data for case 1 in the linear regression the average slope for the patient group would have been even more shallow compared to the best matched healthy controls. [file Image_1.JPEG]

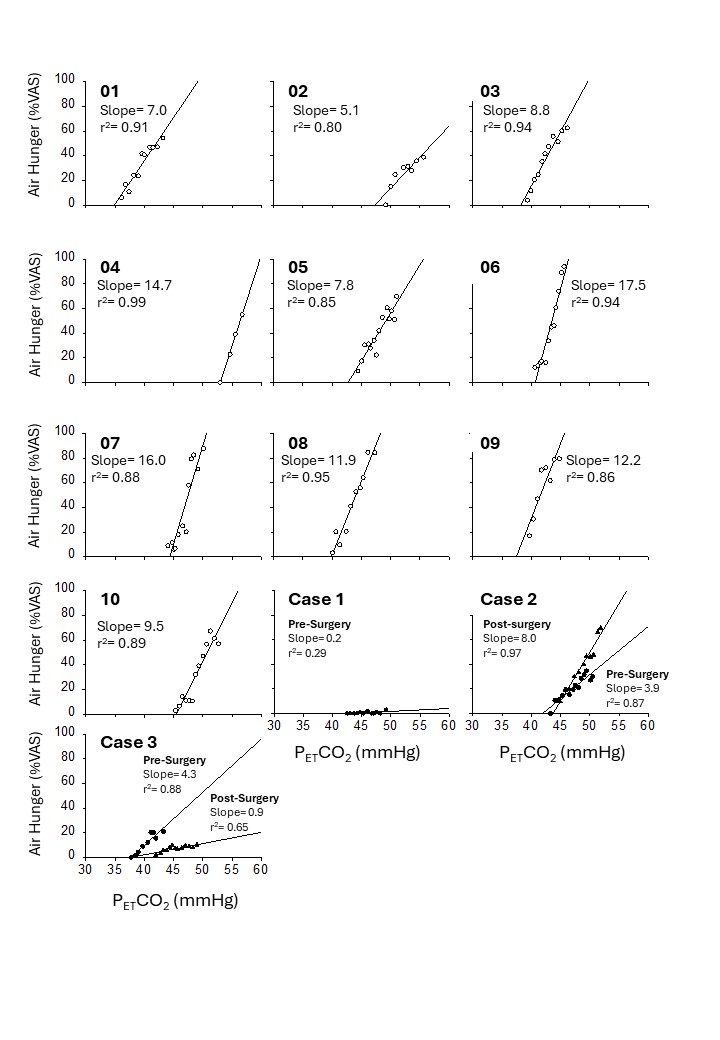

Supplement: SUPPLEMENTARY FIGURE 2 — Individual hypercapnic air hunger response slopes. For the patient group, AH responses for both pre-surgery (closed circles) and post-surgery (triangles) are included. Open circles are used for the healthy controls. [file Image_2.JPEG]
